# Hypothesis: Retinoblastoma protein inactivation mediates effects of histone deacetylase inhibitor-induced Wnt hyperactivation in colorectal cancer cells

**DOI:** 10.7150/jca.37864

**Published:** 2020-01-01

**Authors:** Michael Bordonaro

**Affiliations:** Department of Medical Education, Geisinger Commonwealth School of Medicine, 525 Pine Street, Scranton, PA 18509, USA

**Keywords:** retinoblastoma protein, colorectal cancer, histone deacetylase inhibitor, butyrate, Wnt signaling, apoptosis, cell cycle

## Abstract

Butyrate, a product of dietary fiber and a histone deacetylase inhibitor, induces apoptosis of colorectal cancer cells; this effect of butyrate is in part mediated by its ability to hyperactivate Wnt signaling, and may in part explain the preventive action of dietary fiber against colorectal cancer. However, the mechanisms by which Wnt hyperactivation promotes apoptosis are unknown. Inactivation of the retinoblastoma tumor suppressor occurs in some cancers and can lead to context-dependent cell proliferation or cell death/apoptosis. The function of retinoblastoma protein (Rb) in normal cells is modulation of cell cycle; inactivation of Rb allows for cell cycle progression and, hence, cell proliferation. Wnt signaling is upregulated in a variety of cancers, and deregulated Wnt signaling is a key initiating event in most cases of sporadic colorectal cancer. It has been shown that Wnt signaling activated by APC inactivation can synergize with the inactivation of Rb to induce apoptosis in a manner mediated by increased TORC1 activity, leading to induced metabolic and energy stress. Rb is typically not inactivated in colorectal cancer; however, Rb is phosphorylated and deactivated during cell cycle G1/S transition. This manuscript posits that it is during this time that butyrate/histone deacetylase inhibitor-induced Wnt hyperactivation induces apoptosis in colorectal cancer cells. Thus, the inactivation of Rb in cell cycle progression may synergize with Wnt hyperactivation to induce apoptosis in response to histone deacetylase inhibitors. The hypothesis is that hyperactivation of Wnt signaling enhances colorectal cancer cell apoptosis via the interaction between upregulated Wnt signaling and inactivated Rb during cell cycle progression. This paper discusses this hypothesis and offers initial experimental approaches for testing the hypothesis. A better understanding of how histone deacetylase inhibitors induce colorectal cancer cell apoptosis through hyperactivation of Wnt signaling, and of cross-talk between repression of cell cycle and induction of apoptosis that occurs with treatment with histone deacetylase inhibitors, can assist in the development of novel therapies for colorectal cancer.

## Objective

Reducing morbidity and mortality from colorectal cancer requires a better understanding of cell signaling that mediates sensitivity to agents important for colorectal cancer prevention and treatment. Deregulated Wnt signaling initiates most cases of colorectal cancer and hyperactivation of this signaling by butyrate, a fermentation product of dietary fiber and a histone deacetylate inhibitor, is causally linked to apoptosis and inhibition of cell proliferation. It is unknown how Wnt upregulation causes apoptosis. Given the potential importance of fiber/butyrate for colorectal cancer prevention, and potential uses of histone deacetylase inhibitors against cancer, elucidating the mechanisms whereby Wnt hyperactivation affects cell physiology is significant.

## Fiber, butyrate, and the role of Wnt signaling hyperactivation

Dietary fiber has been shown to be protective against colorectal cancer, and it is thought that this effect is mediated through butyrate, a histone deacetylase inhibitor that is a breakdown product of fiber 1-11. Histone deacetylase inhibitors, which are current or proposed chemotherapeutic agents, induce colorectal cancer cell cycle arrest, differentiation, and/or apoptosis *in vitro* 1,2,12-17. Most colorectal cancers are initiated by deregulated Wnt signaling 18-28, and in these cells histone deacetylase inhibitors hyperactivate Wnt activity 1,2,17; in general, it is known that hyperactivation of oncogenic signaling can induce apoptosis 29. The well-established “just right hypothesis” of colorectal cancer asserts that tumorigenesis is optimally promoted by moderate levels of deregulated Wnt signaling 30. Consistent with this, several studies utilizing genetic activation of Wnt signaling demonstrated that abnormally high levels of Wnt activity induce apoptosis 31-36. Butyrate and other histone deacetylase inhibitors induce colorectal cancer cell apoptosis by both Wnt activity-dependent and Wnt activity-independent mechanisms, and Wnt signaling hyperactivation is causally linked to apoptosis 1,2,17. Whereas Wnt signaling deregulated by mutations leads to moderate levels of Wnt activity and promotes colonic cell proliferation and tumorigenesis, relatively high and relatively low levels of Wnt signaling activity both lead to colorectal cancer cell apoptosis and repressed cell proliferation 1,2,17,37.

Colorectal cancers can develop in individuals with higher levels of fiber intake (6-8), suggesting that butyrate resistance contributes to colonic neoplasia. To study this, a butyrate-resistant colorectal cancer cell line (HCT-R) was developed 2 from butyrate-sensitive HCT-116 colorectal cancer cells exposed to increasing levels of butyrate (up to 5 mM, a physiologically relevant concentration that normally induces colorectal cancer cell apoptosis and repressed growth). HCT-R cells exposed to butyrate exhibit repressed Wnt signaling hyperactivation, lower levels of apoptosis, and increased cell growth compared to parental HCT-116 cells 2. Importantly, HCT-R cells are also cross-resistant to other, structurally distinct, and clinically relevant histone deacetylase inhibitors 2. Thus, for example, HCT-R cells are resistant to the histone deacetylase inhibitor vorinostat 2, evaluated as part of combinatorial therapy against metastatic colorectal cancer 38. Overexpression of Tcf3, which can inhibit Wnt activity, is one contributing factor to butyrate resistance in colorectal cancer cells 39.

## CBP and p300

**Figure 1:**
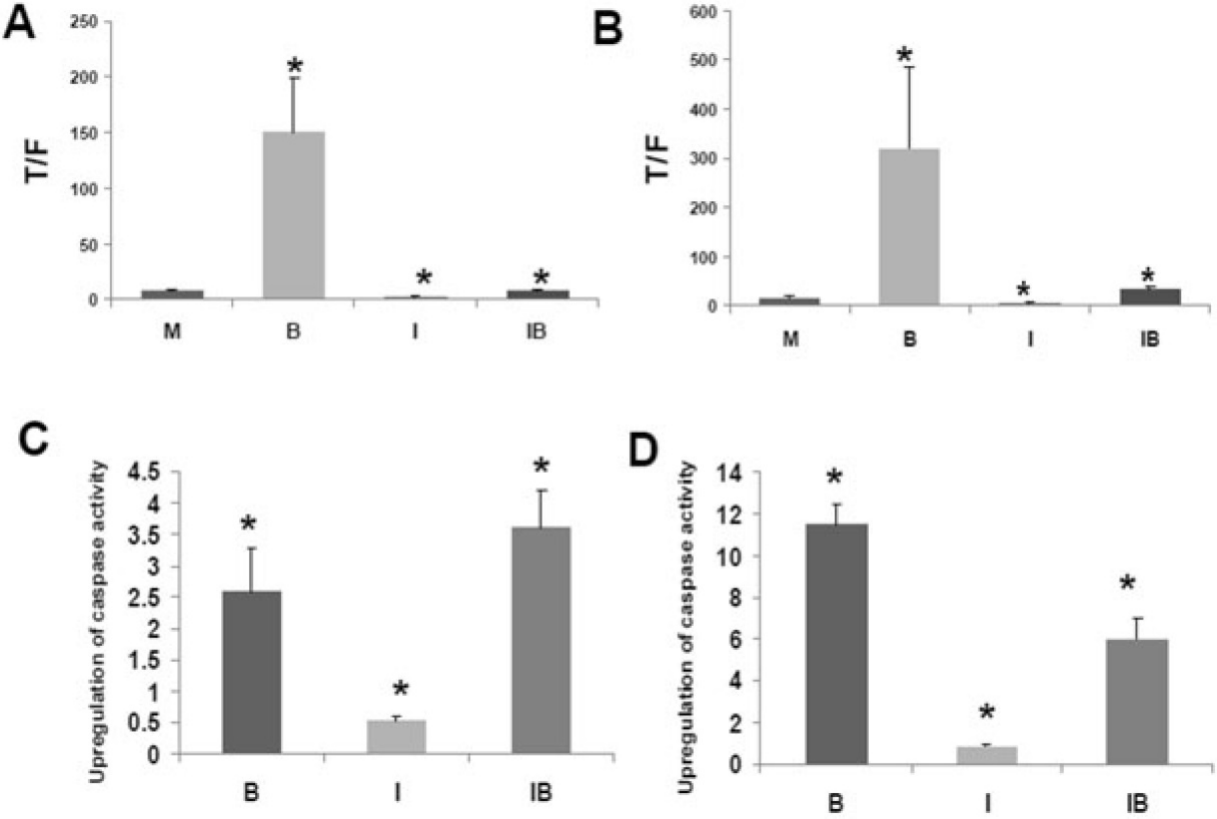
** Effects of butyrate and ICG-001 on Wnt signaling and apoptosis in colorectal cancer cells.** (A) and (C) are from the HCT-116 cell line and (B) and (D) are from SW620 cells. (A) and (B) show Wnt activity as measured by reporter vectors; Wnt activity is hyperactivated by butyrate and suppressed by ICG-001. (C) and (D) show apoptosis as measured by caspase activity; apoptosis is upregulated by butyrate and there are cell-specific effects by ICG-001 on this upregulation. Reproduced from ref. 51, which contains more details about this experiment.

CBP and p300 are important in tumorigenesis, and the association between beta-catenin and CBP or p300 influences Wnt signaling 40-62. The interaction between CBP and Wnt signaling can be dissected utilizing the small molecule inhibitor ICG-001 that binds to CBP but not to p300 40. Treatment of colorectal cancer cells with ICG-001 suppresses the association between CBP and beta-catenin and downregulates Wnt transcriptional activity in colorectal cancer cells 40, while inhibiting colorectal cancer cell proliferation and increasing apoptosis. Data demonstrating both the ability of the histone deacetylase inhibitor butyrate to enhance Wnt signaling in colorectal cancer cells as well as the influence ICG-001 on those metrics are shown (Fig. 1), which is reproduced from the author's previous *Journal of Cancer* publication 51, in support of this manuscript's hypothesis.

## Rb, Wnt hyperactivation, and apoptosis

Inactivation of the retinoblastoma (Rb) tumor suppressor occurs in some cancers and can lead to context-dependent cell proliferation or apoptosis 63. The function of Rb in normal cells is modulation of cell cycle, predominantly through binding to E2F transcription factors. Unphosphorylated (active) Rb typically suppresses cell cycle progression, while inactivating phosphorylation of Rb allows for progression through the cycle and cell proliferation 64. Rb can also have anti-apoptotic functions, possibly through binding to E2F-1; thus, in specific contexts, E2F-1 can stimulate apoptosis rather than proliferation and this action of E2F-1 can be repressed by Rb 65. Hyperactivated Wnt signaling (e.g., from APC knockdown) can synergize with Rb inactivation to induce apoptosis in a manner mediated by increased mTOR activity, leading to induced energy stress and oxidative stress induction 63. The mTORC1 inhibitor rapamycin downregulates apoptosis induced by APC knockdown 63, further suggesting involvement of the mTOR pathway. In addition, a ROS scavenger was able to rescue adherence-independent growth defects in Rb knockdown HCT-116 cells, supporting a role for oxidative stress as a downstream effector of Wnt-Rb inactivation 63.

Rb is typically not mutated in colorectal cancer cells 66. However, during cell cycle G1/S transition Rb is phosphorylated and inactivated 63,64; thus, it is possible that it is during this time that Wnt hyperactivation by histone deacetylase inhibitors induces colorectal cancer cell apoptosis. While Rb knockdown can decrease Wnt signaling, the pro-apoptotic effect of Rb knockdown is due to synergy with the deregulated Wnt activity in these cells, rather than decreased Wnt activity 63. Thus, APC knockdown in beta-catenin mutant HCT-116 cells, which increases Wnt activity, leads to even greater cell death when combined with Rb inactivation 63; that finding suggests that it is the combination of Rb inactivation and increased Wnt activity that induces cell death. However, butyrate also blocks cell cycle, and seems to increase unphosphorylated (active) Rb 67, so there may be competing effects. On the one hand, by enhancing Wnt activity, butyrate may induce apoptosis partially by Wnt hyperactivation during the period of Rb inactivation (G1 to S transition); on the other hand, by blocking cell cycle and increasing hypophosphorylated Rb, butyrate represses the synergy between Wnt activation and Rb inactivation. It has also been shown that p300 interacts with Rb, modulating cell cycle progression in colorectal cancer cells 68.

## Fundamental Hypothesis

**Figure 2:**
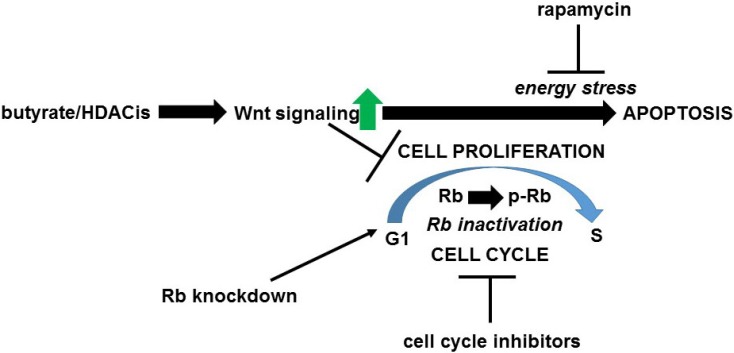
** Proposed hypothesis of Wnt-Rb interactions in response to treatment of neoplastic colonic cells by butyrate/histone deacetylase inhibitors (HDACis)**. Exposure to butyrate and other clinically relevant histone deacetylase inhibitors represses cell cycle progression and upregulate Wnt activity in neoplastic colonic cells; hyperactivated Wnt signaling contributes to enhanced apoptosis (and to repressed cell proliferation). This manuscript posits that the apoptosis induced by butyrate/histone deacetylase inhibitors affects those cells that do not immediately undergo cell cycle arrest and thus progress through the G1 to S transition (which includes the inactivation of Rb by phosphorylation). The combination of hyperactivated Wnt signaling and inactivated Rb induces apoptosis of cells mediated through energy stress (mTOR/TORC1 activity). This enhancement of apoptosis can be repressed by cell cycle inhibitors that block the G1 to S transition and prevent the inactivation of Rb, or by the mTOR inhibitor rapamycin at the level of cellular energy stress. This apoptotic pathway can be promoted by knockdown of Rb expression.

The hypothesis (Fig. 2) is that one mechanism whereby upregulation of Wnt signaling by butyrate enhances colorectal cancer cell apoptosis is the interaction between hyperactivated Wnt signaling and inactivated Rb during cell cycle progression. This hypothesis is consistent with the idea that variation in the levels of Wnt signaling and of Rb inactivation can cause a graded metabolic response 63; thus, when a certain threshold of Wnt activity and Rb inactivation is achieved, metabolic stress is sufficient to induce apoptosis. This manuscript posits that there are two classes of butyrate-treated Wnt activity-positive colorectal cancer cells. The first class of cells are those that are able to go through the G1/S transition in the presence of butyrate-induced Wnt hyperactivation and therefore undergo apoptosis due to the enhanced Wnt activity and inactivated Rb. The second class of cells are those for which butyrate induces cell cycle arrest and Rb hypophosphorylation; these cells would be expected to be temporarily resistant to apoptosis, or would undergo apoptosis by another, Rb-independent mechanism. The differences between these classes of cells may derive from the division of Wnt-positive colorectal cancer cells into high-Wnt and low-Wnt cell fractions, with the former being more sensitive to the pro-apoptotic effects of butyrate 1,2; this would be consistent with a graded apoptotic response to the levels of Wnt activity and of inactivated Rb in given cells 63. Thus, in response to butyrate (or other histone deacetylase inhibitors) high-Wnt cells would be more prone for apoptosis, while the low-Wnt cells would be more likely to undergo cell cycle arrest.

## Testing the hypotheses

The following cell lines would be utilized to test the hypothesis: HCT-116 cells and the F5 p300 knockout line as well as the p300 rescue line in which p300 expression is restored to F5 cells 60, butyrate- resistant HCT-R cells, LT97 colon microadenoma cells that represent the earliest stage of colonic neoplasia from which cells can be isolated 3,4, and the colorectal cancer metastatic line SW620. Normal human colon NCM460 cells will be used as negative controls compared to the Wnt-positive neoplastic colonic cells otherwise evaluated. Histone deacetylase inhibitors used at concentrations that affect Wnt signaling and cell physiology in colorectal cancer cells 2 will be butyrate and the clinically relevant histone diacetylase inhibitors SAHA and LBH589, all of which upregulate Wnt activity and apoptosis, and repress cell proliferation, in colorectal cancer cells; further, butyrate-resistant HCT-R cells are also cross-resistant to histone deacetylase inhibitors other than butyrate 2. We will also briefly consider follow-up *in vivo* experiments utilizing appropriate mouse models.

## Subsection one: What is the role of Wnt activity-Rb cross-talk in the mechanisms whereby histone deacetylase inhibitors affect cell proliferation and apoptosis of neoplastic colonic cells?

It has been shown that inactivation of Rb via mutation or shRNA synergizes with upregulated Wnt activity to induce apoptosis via metabolic (energy) stress, in a manner inhibited by the mTOR inhibitor rapamycin. However, colorectal cancer cells typically express wild-type Rb, yet exhibit enhanced apoptosis associated with hyperactivation of Wnt signaling***.****Sub-hypothesis: the temporary inactivation of Rb as part of the G1 to S transition in proliferating neoplastic colonic cells is one key point at which Wnt hyperactivation (e.g., from histone deacetylase inhibitors) induces apoptosis, in an energy stress-dependent manner. Further, given that this hypothesis requires cell cycle progression for Wnt activity-related apoptosis, we hypothesize competition between cell cycle arrest and apoptosis triggered by exposure to histone deacetylase inhibitors.* Thus, a negative association may exist between butyrate-induced cell cycle arrest and butyrate-induced apoptosis that is dependent upon Rb inactivation during G1/S. Inhibiting the G1 to S transition 69 would be expected to repress histone deacetylase inhibitor-induced apoptosis mediated by Wnt hyperactivation. Mechanisms determining which colorectal cancer cells undergo cell cycle arrest or apoptosis after histone deacetylase inhibitor treatment remain to be identified; however, it has been observed that colorectal cancer cells in culture consist of high-Wnt and low-Wnt fractions that respond to butyrate in a cell type-specific manner 1, and these inherent differences in Wnt activity may influence decisions of cell cycle arrest vs. apoptosis after butyrate treatment.

To evaluate these hypotheses, this study would determine: (a) if Rb knockdown potentiates histone deacetylase inhibitor-induced apoptosis in a Wnt activity-dependent manner, (b) whether inhibiting the G1 to S transition interferes with the ability of histone deacetylase inhibitors to promote colorectal cancer cell apoptosis, (c) whether mTOR activity is required for histone deacetylase inhibitor-induced colorectal cancer cell apoptosis, and (d) how high- and low-Wnt cell fractions influence decisions of cell cycle arrest vs. apoptosis and the role of Rb inactivation and mTOR activity in these processes. To determine whether Rb is required for histone deacetylase inhibitor-induced colorectal cancer cell apoptosis, Rb expression can be knocked out using a stably transfected Rb shRNA expression vector and cells then treated with histone deacetylase inhibitors +/- the Wnt activity inhibitor iCRT3 (to evaluate whether the observed effects are Wnt activity-dependent). Wnt reporter assays 1,2,51-53 would be used to confirm repression of Wnt signaling in the presence of iCRT3. Levels of apoptosis and cell proliferation would be assayed as previously described 51-53. Indole-3-carbinol would be utilized to repress G1 to S transition and Rb phosphorylation 69; indole-3-carbinol, found in *Brassica* vegetables and used as a health supplement, is also in clinical trial for various forms of cancer. Cells can be treated with histone deacetylase inhibitors and apoptosis then measured as described above. mTOR activity can be repressed by treatment with rapamycin 63 or upregulated by shRNA knockdown of TSC2, an mTOR inhibitor 70, cells would be treated with histone deacetylase inhibitors and apoptosis measured as described above. These experiments would underscore the importance of mTOR signaling 63,71,72 in these phenomena. Cells can be separated into low and high Wnt fractions as previously described 1, and Wnt activity levels confirmed by reporter assay 1,2,51-53. High and low Wnt fraction cells would then be treated with histone deacetylase inhibitors and cell proliferation and apoptosis measured. Levels of phosphorylated Rb would be determined in high and low Wnt fractions (+/- histone deacetylase inhibitors) via Western blotting. Wnt activity reporter assays, Western blotting, cell proliferation and apoptosis will be measured as previously described 51-53.

How could *in vivo* experiments be utilized to follow-up the *in vitro* work? The original work concerning Wnt-Rb interactions 63 utilized *Drosophila* models and it is certainly possible to adapt some of the abovementioned experimental work in that model. More pertinent for potential human utility of these studies would be experimental follow-up utilizing mouse models. The simplest approach to begin *in vivo* work on this hypothesis is to utilize immunodeficient mice with colorectal cancer cell xenografts, treated as above (e.g., stable Rb knockdown; use of histone deacetylase inhibitors, rapamycin, indole-3-carbinol, etc.). A more powerful approach would be to utilize transgenic mouse models. The typical embryonic lethality observed with Rb-/- mice can be overcome through the use of tissue-targeted conditional Rb knockout mice 73. Crossing such mice with various *APC* strains of mutant mice will create *in vivo* models that couple Wnt hyperactivation with Rb knockout targeted to the intestine and activated at varied times during neoplastic development. These mice can be treated in an analogous fashion to the *in vitro* work (histone deacetylase inhibitors, rapamycin, indole-3-carbinol, etc.).

## Expected results for testing section one of our hypothesis

It is expected that cotreatment of colorectal cancer cells with histone deacetylase inhibitors and indole-3-carbinol 70 would suppress histone deacetylase inhibitor-mediated apoptosis, as would treatment with rapamycin. The indole-3-carbinol results would suggest that this compound, an anti-cancer therapeutic agent, may interfere with histone deacetylase inhibitor-induced apoptosis. On the other hand, Rb knockdown would be expected to enhance histone deacetylase inhibitor-mediated apoptosis in a rapamycin-sensitive manner, confirming involvement of mTOR in the apoptotic pathway. Thus, upregulation of TORC1 via TSC2 knockdown would also be expected to enhance histone deacetylase inhibitor-mediated apoptosis. In all cases where apoptosis induced by histone deacetylase inhibitors is upregulated, this effect is expected to be abrogated by inhibition of Wnt signaling by iCRT3. High Wnt activity cells are expected to demonstrate a greater propensity toward undergoing apoptosis rather than cell cycle arrest when exposed to histone deacetylase inhibitors compared to low Wnt activity cells, and this greater propensity to apoptosis would be sensitive to perturbations of Wnt-Rb crosstalk, such as iCRT3 (repressing Wnt activity) and rapamycin (repressing mTOR activity). It is expected that cotreatment of colorectal cancer cells with histone deacetylase inhibitors and indole-3-carbinol 70, would suppress histone deacetylase inhibitor-mediated apoptosis, as would rapamycin, given the importance of mTOR activity 71,72. On the other hand, Rb knockdown would be expected to enhance butyrate-mediated apoptosis, and this enhancement would be inhibited by rapamycin.

With respect to the proposed *in vivo* work, one would expect that histone deacetylase inhibitors would enhance the apoptosis observed in *Drosophila* cells exhibiting Wnt hyperactivation and Rb knockdown. In xenograft mouse models, the transplanted human colorectal cancer cells would be expected to be sensitized to apoptosis induced by histone deacetylase inhibitors via Rb knockdown (e.g., by stably expressed Rb shRNA). This sensitization would be expected to be reversed by rapamycin or indole-3-carbinol. With respect to transgenic mouse models, intestine-targeted conditional knockdown of Rb in *APC* mutant mice would be expected to enhance apoptosis of the neoplastic cells, coupled to sensitization to histone deacetylase inhibitors (which, again, could be reversed by rapamycin or indole-3-carbinol).

## Subsection two: Can the association between Wnt activity, p300, and Rb inactivation be modulated to reverse butyrate/histone deacetylase inhibitor-resistance in neoplastic colonic cells?

Colorectal cancer cells can become resistant to butyrate and other histone deacetylase inhibitors, and loss of p300 activity is associated with butyrate resistance in the HCT-116 colorectal cancer cell line 52. HCT-116 cells are normally characterized by interactions between p300 and Rb that control the G1 to S transition in these cells 68; p300 also interacts with beta-catenin to modulate Wnt hyperactivation 52. These findings suggest the sub-hypothesis that *p300 activity is required for the competitive balance between cell cycle arrest and apoptosis in histone deacetylase inhibitor-treated colorectal cancer cells, and that butyrate resistance associated with p300 loss is in part due to aberrant Wnt activity-Rb cross-talk during cell cycle progression. Further, this manuscript hypothesizes that the accelerated G1 to S transition due to p300 deficiency interferes with mTOR activity normally observed in late G1, thus reducing energy stress and apoptosis.*

**Figure 3:**
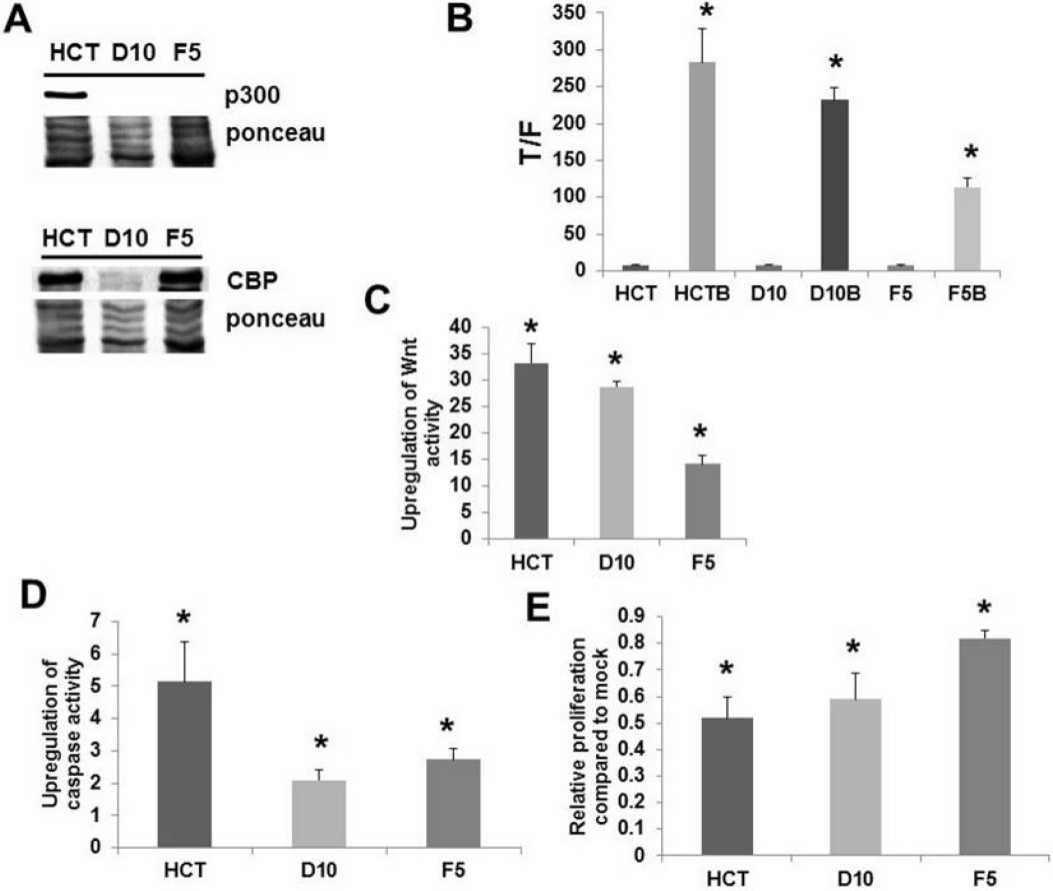
** Knockout of p300 affects response of colorectal cancer cells to butyrate.** (A) Expression of p300 as assayed by Western Blot in HCT-116 cells and in two p300 knockout versions of this cell line. (B) Wnt signaling assayed as described in Fig. 1, showing decreased hyperactivation of Wnt signaling by butyrate in the knockout lines, (C) the fold up-regulation of Wnt activity, (D) apoptosis measured as described in Fig. 1; the knockout lines demonstrate decreased upregulation of apoptosis by butyrate, (E) cell proliferation measured as described in ref. 74; the knockout lines are more resistant to the effects of butyrate on cell growth. Reproduced from ref. 74, which contains more details about this experiment.

Rb associates with p300 during cell cycle to modulate the G1 to S transition; it is thought that p300 inhibits Rb phosphorylation in early G1 to prevent premature entry into S phase; on the other hand, p300, and possibly CBP, stimulates Rb phosphorylation in late G1 to promote transition to S phase at the proper time 68. p300 knockout HCT-116 cells exhibit defects in cell proliferation, with slower doubling times than wild-type cells; paradoxically, these knockout cells display aberrant cell cycle dynamics characterized by a greater proportion of S phase and lower proportion of G1 phase cells compared to wild type cells 68. It has been shown 74 that p300 knockout HCT-116 cells exhibit butyrate resistance (Fig. 3, from 74); consistent with the loss of p300 expression observed in the HCT-R line 52, a butyrate-resistant colorectal cancer cell line derived from HCT-116 cells exposed to increasing levels of butyrate over time 2. HCT-R cells also grow slower than the parental HCT-116 line [2, unpublished data]; p300 knockout cells demonstrate premature G1 to S transition and Rb hyperphosphorylation, upon release from serum depletion-induced cell cycle blockade 68. p300 affects Wnt hyperactivation by butyrate 52. These findings suggest that p300 influences Wnt activity-Rb cross-talk that controls decisions of cell cycle arrest or apoptosis upon exposure to histone deacetylase inhibitors. While we hypothesize that G1 to S transition and Rb inactivation are in part required for histone deacetylase inhibitor-induced apoptosis in neoplastic colonic cells, we also posit that aberrant cell cycle in p300-deficient colorectal cancer cells, including premature Rb inactivation and accelerated entry into S phase, inhibits the energy stress required for apoptosis.

**Figure 4:**
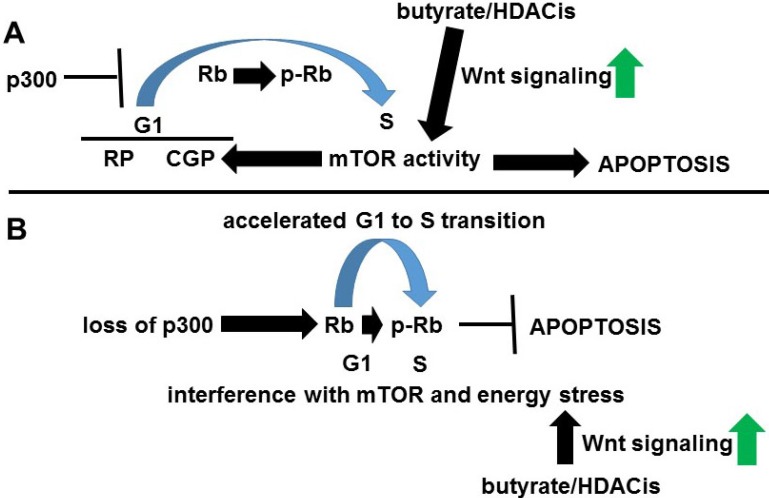
** Role of p300 in Wnt activity-Rb crosstalk**. (A) In normal circumstances, p300 sufficiently inhibits the G1 to S transition to allow normal mTOR activity, resulting in energy stress-induced apoptosis upon Wnt hyperactivation coupled to Rb inactivation. Wnt signaling can also increase mTOR activity. (B) Loss of p300 results in premature G1 to S transition, interfering with normal mTOR activity and repressing histone deacetylase inhibitor-induced apoptosis.

G1 phase has two control points influencing cell cycle; first, the “restriction point” and a second, later “cell growth point” that is modulated by mTOR activity 71. At this second step, mTOR inhibits TGF-β signaling; this inhibition reduces p27 expression and allows cyclin E-CDK2 to promote S phase 70. Therefore, a series of defined steps during G1 to S transition, including mTOR activity, may be required for histone deacetylase inhibitor-induced apoptosis; these steps are deregulated by p300 deficiency 68, possibly leading to butyrate resistance (Fig. 4). While it is possible that the lack of p300 interferes with Rb phosphorylation in late G1, CBP likely compensates, since p300 knockout cells efficiently enter S phase 68.

It is also known that Wnt signaling increases mTOR activity, which can potentiate histone deacetylase inhibitor-induced apoptosis in the presence of p300, as histone deacetylase inhibitors hyperactivate Wnt signaling (stimulating mTOR and synergizing with Rb inactivation to enhance mTOR energy stress). Interestingly, while p300 knockout cells exhibit a proliferation defect under normal culture conditions, they grow better than wild-type cells under serum starvation, exhibiting continued hyperphosphorylation of Rb 68.

## To evaluate the second section of our hypothesis of Subsection two we would

(a) Determine whether the p300 status of butyrate-sensitive and butyrate-resistant colorectal cells effects levels of active vs. inactivated Rb, mTOR activity, and superoxide (a measure of oxidative stress). This study will also determine whether p300 knockout cells proliferate in serum-deprived conditions as previously reported 68, and if response to histone deacetylase inhibitors is altered under these conditions, measuring levels of apoptosis and cell proliferation as well as levels of phosphorylated, inactivated Rb (p-Rb), mTOR activity, and superoxide.

(b) Determine whether p300 deficiency, or the balance between CBP-Wnt and p300-Wnt signaling, alters the balance between butyrate-induced cell cycle arrest vs. apoptosis to produce butyrate resistance, and whether this effect is rapamycin-sensitive, indicative of mTOR involvement, and exhibits cell type-specific effects on butyrate-induced apoptosis and proliferation arrest 51-53; these effects may in part be due to differences in levels of active vs. inactive Rb in these cells, together with the levels of Wnt hyperactivation observed with ICG-001-butyrate cotreatment. This study will determine whether the cell type-specific effects of ICG-001 and butyrate cotreatment of colorectal cancer cells on apoptosis and cell proliferation are affected by the cross-talk between hyperactivated Wnt signaling and Rb inactivation during cell cycle, mediated by mTOR signaling in a rapamycin-sensitive manner.

(c) Evaluate whether upregulation of mTOR/ TORC1 activity can reverse butyrate resistance by inducing energy stress despite premature entry into S phase.

These more molecular and mechanistic experiments will be more difficult to extend to *in vivo* models compared to that of subsection one. However, utilization of YH249, a specific inhibitor of p300-Wnt signaling 61, will allow for the role of p300 to be dissected in both mouse xenograft and transgenic mouse models (described above) of the Wnt-Rb interaction. Further, water soluble analogs of the specific CBP-Wnt inhibitor ICG-001, already tested in mouse models of colorectal cancer 40, can be utilized to understand how Wnt-Rb cross-talk influences effects of butyrate-ICG-001 combinatorial treatment.

## Expected results for testing two of our hypothesis

Given that (1) it is hypothesized that histone deacetylase inhibitor-induced colorectal cancer cell apoptosis is mediated by the Wnt-Rb-mTOR pathway; (2) Wnt hyperactivation (and its consequences) has CBP- and p300-mediated components 51-53; and (3) it is hypothesized that p300 and its activity is essential for efficient induction of apoptosis through Wnt hyperactivation together with Rb inactivation and mTOR/TORC1 activity; it is expected that:

(a) p300 knockout cells will exhibit reduced levels of p-RB, mTOR/TORC1 activity, and superoxide/ROS, both with respect to untreated cells and particularly after treatment with histone deacetylase inhibitors, concomitant with resistance to the apoptotic effects of butyrate and other histone deacetylase inhibitors. It is expected that under serum-deprived conditions, p300 knockout cells will exhibit a more pro-proliferative phenotype compared to wild-type HCT-116 cells under the same conditions and will thus become more sensitive to the apoptotic-inducing effects of histone deacetylase inhibitors that depend upon Rb inactivation in cell cycle progression. It is expected that in all cases, p300 rescue cells will resemble wild-type HCT-116 cells, confirming the importance of p300 in these phenomena. In addition, the observed cell type differences are expected to be reduced or abrogated by rapamycin treatment, confirming dependence on mTOR activity. Finally, energy stress will be demonstrated by a correlation between increased apoptosis and increased levels of superoxide/ROS as measured by the DHE assay.

(b) Cell type-specific effects of ICG-001 and butyrate cotreatment of colorectal cancer cells on apoptosis and cell proliferation will be dependent upon the cross-talk between hyperactivated Wnt signaling and Rb inactivation during cell cycle, and will be mediated by mTOR signaling, and thus abrogated by rapamycin treatment.

(c) Upregulation of mTOR/TORC1 activity will at least in part restore sensitivity to butyrate/histone deacetylase inhibitors in HCT-R and p300 knockout cells.

For all subsections, it is uncertain whether colon microadenoma LT97 cells will be, to a greater or lesser extent, dependent upon the Wnt-Rb-mTOR pathway for histone deacetylase inhibitor-induced apoptosis than are colorectal cancer cells, including metastatic SW620 cells. On the one hand, LT97 cells exhibit slower proliferative dynamics than do colorectal cancer cells 3, suggesting a lesser dependence on apoptotic mechanisms involving cell cycle progression and Rb inactivation; on the other hand, LT97 cells are more sensitive to the effects of the histone deacetylase inhibitor butyrate (proliferation and apoptosis) 4,53, so the overall influence of Wnt-Rb-mTOR on LT97 physiology is unpredictable and must be empirically determined.

In all cases (and for all subsections), normal NCM460 cells are expected to lack the Wnt-Rb crosstalk responsible for promoting histone deacetylase inhibitor-induced apoptosis.

With respect to mouse models, it is expected that the p300-Wnt inhibitor YH249 will inhibit the enhancement of apoptosis (basal and that induced by histone deacetylase inhibitors) that results from Wnt hyperactivation coupled with Rb inactivation/ knockdown. The ability of butyrate combined with an ICG-001 analog to induce cell death *in vivo* will be dependent upon Wnt hyperactivation coupled with Rb inactivation/knockdown, and will be inhibited by rapamycin, suggesting that these effects are mediated by mTOR signaling.

## Epigenetic regulation

The main focus of this manuscript is an extension of the discovery 63 that Rb inactivation synergizes with Wnt hyperactivation to drive apoptosis. Thus, even though histone deacetylase inhibitors do have Wnt-independent mechanisms of action, it will be the Wnt-dependent functions that are likely affected by Rb status, and it is this hypothesis that is evaluated by the experiments proposed above. Also, the effects of histone deacetylase inhibitors (including butyrate) on Wnt signaling involves epigenetic regulation, via altered expression of relevant genes, and the opening up of chromatin to access beta-catenin-Tcf complexes and cotranscriptional factors sch as CBP and p300, themselves epigenetic regulators. Therefore, the epigenetic regulation functions of histone deacetylase inhibitors are fully integrated into our hypothesis, and are not separate from it. Further, the importance of the histone acetylase p300 to our hypothesis in subsection two necessarily incorporates epigenetic regulation (i.e., histone acetylation) in what is proposed.

As stated, a component of the apoptosis (and cell growth inhibition) promoted by histone deacetylase inhibitor treatment of colorectal cancer cells will be Wnt-independent. Thus, even though inhibition of Wnt signaling significantly decreases apoptosis and growth arrest induced by histone deacetylase inhibitors, apoptosis and growth arrest are not eliminated 1. Therefore, a fraction of the apoptotic effect observed in such cells will derive from Wnt-independent actions of histone deacetylase inhibitors, most likely through the epigenetic regulation of gene expression via net increased histone acetylation. Wnt-independent effects of histone deacetylase inhibitors can indirectly influence Wnt-Rb cross-talk through altered expression of factors that mediate these pathways and their downstream consequences.

Therefore, undoubtedly, the expected cellular phenotypes will be affected by the epigenetic regulation and consequent changes in gene expression due to treatment with histone deacetylase inhibitors. However, the specific hypothesis addressed in this manuscript directly focuses on the Wnt-dependent aspects of this regulation, mediated through Rb inactivation, leading to metabolic and energy stress and apoptosis, dependent upon mTOR signaling. Wnt-independent epigenetic regulation is relevant insofar as it influences Wnt-Rb cross-talk in the manner described above.

## Conclusion

Successful completion of these proposed studies will provide a better understanding of (a) how butyrate and other histone deacetylase inhibitors induce colorectal cancer cell apoptosis through hyperactivation of Wnt signaling, and (b) cross-talk between repression of cell cycle and induction of apoptosis that occurs with treatment with histone deacetylase inhibitors. These findings can allow for (a) optimization of histone deacetylase inhibitors as preventive and therapeutic agents, respectively against colorectal cancer, and (b) provide for approaches to prevent or reverse resistance to the effects of butyrate/histone deacetylase inhibitors on neoplastic colonic cells. Countering butyrate/histone deacetylase inhibitor resistance would allow the full potential of histone deacetylase inhibitor therapy to be achieved against colorectal cancer. Thus, the findings generated by testing this hypothesis are likely to determine how synergy between Wnt hyperactivation and Rb inactivation, mediated by cell cycle-apoptosis cross-talk, can be leveraged for anti-colorectal cancer therapeutics. While not yet widely utilized for colorectal cancer treatment, histone deacetylase inhibitor colorectal cancer clinical trials have been conducted and at least one trial is ongoing. With respect to fiber and butyrate, while the effect of fiber on prevention is likely not solely due to butyrate, our work strongly suggests that butyrate is a crucial component of the preventative action of fiber. An additional objective of evaluating our hypothesis is to stimulate increased interest in the utility of histone deacetylase inhibitors in colorectal cancer prevention/therapy. In summary, a research project based on this hypothesis can impact future treatment options by demonstrating how histone deacetylase inhibitors can be more effectively utilized against colorectal cancer and may influence prevention strategies through a better understanding of the mechanisms of butyrate resistance.

## Abbreviations

Rb: retinoblastoma protein
HDACis: histone deacetylase inhibitors
